# Neoadjuvant chemotherapy followed by interval debulking surgery for advanced epithelial ovarian cancer: GOTIC-019 study

**DOI:** 10.1007/s10147-023-02329-7

**Published:** 2023-05-04

**Authors:** Shoji Nagao, Jun Tamura, Takashi Shibutani, Maiko Miwa, Tomoyasu Kato, Ayumi Shikama, Yuji Takei, Natsuko Kamiya, Naoki Inoue, Kazuto Nakamura, Aya Inoue, Koji Yamamoto, Keiichi Fujiwara, Mitsuaki Suzuki

**Affiliations:** 1 Department of Obstetrics and Gynecology, Faculty of Medicine, Dentistry and Pharmaceutical Sciences, Okayama University, 2-5-1 Shikata-cho, Kita-ku, Okayama, Japan; 2 Department of Biostatistics, Yokohama City University School of Medicine, 3-9 Fukuura, Kanazawa-ku, Yokohama, Japan; 3 Department of Gynecologic Oncology, Hyogo Cancer Center, 13-70 Kitaoji-cho, Akashi, Hyogo 673-8558 Japan; 4 Department of Gynecologic Oncology, Saitama Medical University International Medical Center, Yamane, Hidaka, 1397-1 Japan; 5 Department of Gynecologic Oncology, National Cancer Center Hospital, 5-1-1 Tsukiji, Chuo-ku, Tokyo, Japan; 6 Department of Obstetrics and Gynecology, Graduate School of Comprehensive Human Sciences, University of Tsukuba, 1-1-1 Tennoudai, Tsukuba, Japan; 7 Department of Obstetrics and Gynecology, Jichi Medical University, 3311-1 Yakushiji, Shimotsuke, Japan; 8 Department of Obstetrics and Gynecology, Yokohama City University School of Medicine, 3-9 Fukuura, Kanazawa-ku, Yokohama, Japan; 9 Department of Obstetrics and Gynecology, Gunma University, 3-39-15 Showa-cho, Maebashi, Japan; 10 Department of Gynecologic Oncology, Gunma Prefectural Cancer Center, 617-1 Takabayashi-cho, Ota, Japan; 11 Department of Obstetrics and Gynecology, Ehime University School of Medicine, 454 Shitsukawa, Toon, Japan; 12 Department of Obstetrics and Gynecology, Shin-Yurigaoka General Hospital, 255 Furusawatsuko, Asao-ku, Kawasaki, Japan

**Keywords:** Neoadjuvant chemotherapy, Epithelial ovarian cancer, Adjuvant chemotherapy, Interval debulking surgery, Primary debulking surgery

## Abstract

**Introduction:**

Three randomized controlled trials have resulted in extremely extensive application of the strategy of using neoadjuvant chemotherapy (NAC) followed by interval debulking surgery (IDS) for patients with advanced epithelial ovarian cancer in Japan. This study aimed to evaluate the status and effectiveness of treatment strategies using NAC followed by IDS in Japanese clinical practice.

**Patients and methods:**

We conducted a multi-institutional observational study of 940 women with Federation of Gynecology and Obstetrics (FIGO) stages III–IV epithelial ovarian cancer treated at one of nine centers between 2010 and 2015. Progression-free survival (PFS) and overall survival (OS) were compared between 486 propensity-score matched participants who underwent NAC followed by IDS and primary debulking surgery (PDS) followed by adjuvant chemotherapy.

**Results:**

Patients with FIGO stage IIIC receiving NAC had a shorter OS (median OS: 48.1 vs. 68.2 months, hazard ratio [HR]: 1.34; 95% confidence interval [CI] 0.99–1.82, p = 0.06) but not PFS (median PFS: 19.7 vs. 19.4 months, HR: 1.02; 95% CI: 0.80–1.31, p = 0.88). However, patients with FIGO stage IV receiving NAC and PDS had comparable PFS (median PFS: 16.6 vs. 14.7 months, HR: 1.07 95% CI: 0.74–1.53, p = 0.73) and OS (median PFS: 45.2 vs. 35.7 months, HR: 0.98; 95% CI: 0.65–1.47, p = 0.93).

**Conclusions:**

NAC followed by IDS did not improve survival. In patients with FIGO stage IIIC, NAC may be associated with a shorter OS.

## Introduction

Primary debulking surgery (PDS) followed by platinum-based chemotherapy is the standard treatment for patients with epithelial ovarian cancer [1]. The maximum cytoreduction in PDS has a major positive effect on survival; however, because > 50% of patients with epithelial ovarian cancer have wide tumor dissemination and/or distant metastasis, optimal or complete surgery can be achieved in only 25%–40% of patients undergoing PDS [2–4].

Recently, the EORTC55971 and CHORUS studies demonstrated that neoadjuvant chemotherapy (NAC) followed by interval debulking surgery (IDS) was non-inferior to PDS followed by adjuvant chemotherapy in terms of survival of patients with Federation of Gynecology and Obstetrics (FIGO) stage IIIC or IV epithelial ovarian cancer [5–7]. Although the JCOG0602 trial, which was conducted in Japan, could not demonstrate the non-inferiority of NAC, the median overall survival (OS) of patients receiving PDS and NAC was 49.0 vs. 44.3 months, respectively; as the study had a smaller sample size, the non-inferiority noted in previous studies could not be denied [8]. These studies revealed that, compared with the strategy using PDS, NAC achieved a high complete surgery rate in IDS and decreased the complication rate [5, 6, 8]. This evidence prompted many patients with apparently unresectable tumors, low performance status, or medical complications to undergo NAC followed by IDS in Japan.

However, the extensive use of NAC may have an adverse effect on survival. First, a pooled analysis of the EORTC55971 and the CHORUS studies suggested that patients with FIGO stage IIIC disease and whose largest metastatic tumor size was < 5 cm had a significant progression-free survival (PFS) advantage if treated with PDS [7]. Second, patients who received NAC appeared to benefit from subsequent IDS only when complete resection was achieved [5, 8]. Third, the response to NAC using a platinum-based regimen depends on the histological type and biological status [1]. Patients with ovarian cancer who do not respond to NAC may lose the opportunity to receive IDS. The application of a reasonable NAC strategy is essential.

This study aimed to evaluate the actual status and effectiveness of treatment strategies using NAC followed by IDS in Japanese clinical practice. Additionally, we examined the effect of residual tumors on patient survival.

## Materials and methods

### Patient recruitment

Consecutive patients with histologically confirmed FIGO stage (2014) III or IV epithelial ovarian, tubal, or primary peritoneal cancer who initiated primary treatment between January 2010 and December 2015 were registered. We used clinical staging using radiological imaging in patients not deemed suitable for surgical assessment. Patients who underwent diagnosis of malignancy only by cytology of ascitic fluid were included if the presence of a malignant epithelial tumor was confirmed. Patients who had received no more than two cycles of platinum-based chemotherapy or who had borderline tumors or other invasive cancers were excluded.

### Study design

This retrospective cohort study compared the PFS and OS of patients who received NAC with those who received PDS. The initial approach to advanced epithelial ovarian cancer has been aggressive cytoreduction with PDS, such as bilateral salpingo-oophorectomy, hysterectomy, omentectomy, pelvic and/or para-aortic lymphadenectomy, resection of either the small or large intestine, splenectomy, diaphragmatic stripping, or other related procedures. In considerable number of patients, we were not able to determine whether a surgeon had planned a primary surgery for maximum debulking or not. Therefore, we defined the minimum procedure of primary surgery for tumor resection (not biopsy) as bilateral salpingo-oophorectomy and omentectomy; patients who received these procedures before chemotherapy were included in the PDS group. Those who did not receive the minimum procedure before chemotherapy were included in the NAC group, regardless of whether primary surgery was performed. In addition, patients diagnosed by cytology alone and started chemotherapy were also included in the NAC group.

### Outcomes

PFS was measured from the date of PDS or the start date of NAC to the date of radiographic relapse, progression, death from all causes, or last contact for disease-free patients. OS was defined as the period from the date of PDS or the start date of NAC to death or the date of last contact. Regarding surgical outcome, complete surgery, optimal surgery, and suboptimal surgery were defined macroscopically as no residual tumor, a residual tumor < 1 cm in maximum diameter, and a residual tumor ≥ 1 cm at the end of the procedure, respectively.

### Data collection

Data regarding the following clinicopathological parameters were collected for subsequent analysis: (1) age, height, weight, and performance status at the initiation of primary therapy; (2) FIGO stage (restaged according to FIGO2014); (3) histologic type and grade; (4) presence of thrombosis; (5) accumulation of ascites and a history of paracentesis; (6) the value of CA125 and carcinoembryonic antigen (CEA) at the initiation of primary therapy and at the end of NAC; (7) date of surgery; (8) surgical procedure received; (9) maximum size of the residual tumor; (10) initial date of chemotherapy; (11) chemotherapy regimen and number of cycles received; (12) date of subsequent radiologic relapse or progression; (13) date of death or last contact; and (14) cause of death.

### Statistical analyses

Propensity-score matching was performed to minimize confounding bias and balance the variables [9]. Propensity scores for the probability of the NAC group were estimated using a logistic regression model with PS, FIGO stage, presence of thrombosis, ascites retention, and age as covariates. A 1:1 nearest neighbor algorithm of caliper with a 0.2 standard deviation of the logit of the propensity score was used for matching. Propensity-score matching was performed using the MatchIt R package. The impact of surgical outcomes on survival was analyzed for all patients rather than matching cohorts, because it is influenced by variables other than the covariates used to estimate the propensity score, which were determined prior to treatment initiation. The Mann–Whitney U test and chi-square test were used to compare the background information of each group. OS and PFS curves were analyzed using the Kaplan–Meier method, and Cox regression models were performed. Statistical tests were performed at the α = 0.05 (2-sided) level of statistical significance using the R statistical package (R Foundation for Statistical Computing).

### Study registration

The protocol was approved by the Gynecologic Oncology Trial and Investigation Consortium (GOTIC) protocol committee in September 2018. The study was registered immediately with the University Hospital Medical Information Network (http://www.umin.ac.jp, No. 000036816). The study was approved by the Institutional Review Board of each participating institution before registration initiation and was conducted according to Declaration of Helsinki. This report was prepared in accordance with the STROBE statement [10].

## Results

**Fig. 1:**
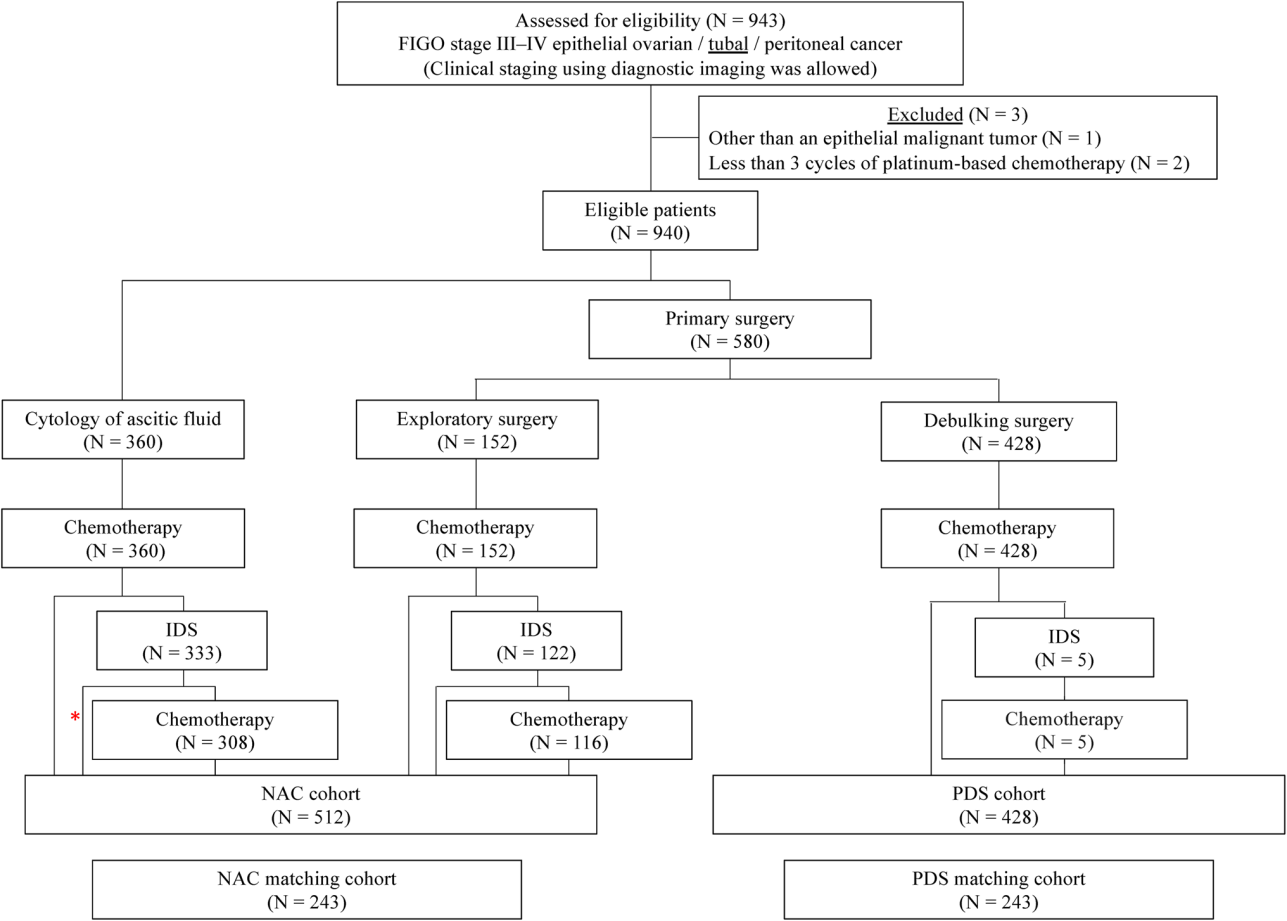
Flow diagram of patient inclusion. *PDS* primary debulking surgery, *IDS* interval debulking surgery, *SDS* secondary debulking surgery, *NAC* neoadjuvant chemotherapy.*One patient, who received NAC followed by IDS administered only day 1 of cycle 1 of weekly TC therapy

Overall, 943 patients with FIGO stage III to IV epithelial ovarian, tubal, or peritoneal cancer were registered from nine centers participating in the GOTIC (Fig. 1). Among the total 940 patients, excluding 3 patients who did not meet the eligibility criteria, 428 (45%) were assigned to the PDS cohort and the remaining 512 (55%) were assigned to the NAC cohort. The adoption rate of NAC varied across the institutions from 21 to 67%. Propensity-score matching yielded 243 pairs from both the NAC and PDS cohorts.

Table 1 shows the patient characteristics in terms of the NAC and PDS cohorts. The median age was slightly higher in the NAC group (62 vs. 59 years). Patients with poor performance status (≥ 2; n = 117/187, 63%), high-grade serous carcinoma (n = 358/596, 60%), ascites (n = 433/716, 61%), thrombotic disease (n = 102/159, 64%), and those who required paracentesis for symptom relief (n = 195/266, 73%) tended to receive NAC. The median CA125 level was significantly higher in the NAC group versus the PDS group (1269 vs. 498 U/mL).

**Table 1:** Patient characteristics

	NAC cohort (N = 512)	PDS cohort (N = 428)	p
Age (median, range)	62 (29–85)	59 (20–89)	< 0.001^*^
PS			
0	218 (43%)	225 (53%)	
1	177 (35%)	133 (31%)	
2–	117 (22%)	70 (16%)	0.0015^**^
FIGO stage			
IIIA–B	26 (5%)	132 (31%)	
IIIC	290 (57%)	205 (48%)	
IV	196 (38%)	91 (21%)	< 0.001^**^
Histology			
HGSC	358 (70%)	238 (56%)	
Endometrioid	15 (3%)	39 (9%)	
Clear cell	28 (5%)	82 (19%)	
Mucinous	7 (1%)	18 (4%)	
LGSC	8 (2%)	7 (4%)	
Adenocarcinoma	21 (4%)	3 (1%)	
Carcinoma	62 (12%)	10 (2%)	
Others	13 (3%)	31 (5%)	< 0.001^**^
CA125 (U/mL) (median, range)	1269 (12–84,271)	498 (2–28,620)	< 0.001^*^
Ascites			
Paracentesis	195 (38%)	71 (17%)	
Present	238 (47%)	212 (50%)	
Absent	78 (15%)	142 (33%)	
Unclear	1 (0%)	3 (1%)	< 0.001^**^
Thrombosis			
None	409 (80%)	371 (87%)	
DVT	70 (14%)	36 (8%)	
PE	31 (6%)	19 (4%)	
Others	1^a^ (0%)	2^b^ (1%)	
Unclear	1 (0%)	0 (0%)	0.034^**^

*Mann–Whitney U-test, **chi-square test

^a^Unspecified thrombosis, ^b^One cerebral infarction and one ovarian vein thrombosis

*NAC* neoadjuvant chemotherapy, *PDS* primary debulking surgery, *HGSC* high-grade serous carcinoma, *LGSC* low-grade serous carcinoma, *DVT* deep venous thrombosis, *PE* pulmonary embolism

### Chemotherapy

Table 2 shows the chemotherapy details of both the NAC and PDS groups. Patients underwent a median of four NAC cycles, and 364 patients (71%) received three to five cycles of NAC. Patients in the NAC group received a median of three adjuvant chemotherapy cycles, while those in the PDS group received six. About half of the patients (55%) in the PDS group received six cycles of chemotherapy after PDS.

**Table 2:** Chemotherapy

	NAC cohort (N = 512)	PDS cohort (N = 428)
Neoadjuvant chemotherapy		
Regimen		
TC	184 (36%)	–
DC	18 (4%)	–
Dose-dense TC	216 (42%)	–
Weekly TC	25 (5%)	–
Dose-dense TCip	40 (8%)	–
TC + Bev	5 (1%)	–
DC + Bev	2 (0%)	–
Others	22 (4%)	–
Cycle number		
0	0	–
1–2	16 (3%)	–
3	99 (19%)	–
4	196 (38%)	–
5	69 (13%)	–
6	98 (19%)	–
7–	34 (7%)	–
Adjuvant chemotherapy		
Regimen		
TC	141 (28%)	166 (39%)
DC	27 (5%)	18 (4%)
Dose-dense TC	171 (33%)	109 (25%)
Weekly TC	26* (5%)	9 (2%)
Dose-dense TCip	27 (5%)	40 (9%)
TC + Bev	13 (3%)	68 (16%)
Others	18 (4%)	18 (4%)
Not done	89 (17%)	–
Cycle number		
0	89* (17%)	–
1–2	75 (15%)	–
3	170 (33%)	72 (17%)
4	104 (20%)	26 (6%)
5	21 (4%)	23 (5%)
6	38 (7%)	236 (55%)
7–	15 (3%)	71 (17%)

*TC* paclitaxel + carboplatin, *DC* docetaxel + carboplatin, Dose-dense TC dose-dense paclitaxel + carboplatin, *Weekly TC* weekly paclitaxel + weekly carboplatin, *Dose-dense TCip* dose-dense paclitaxel + intraperitoneal carboplatin, *TC + Bev* paclitaxel + carboplatin + bevacizumab + bevacizumab maintenance therapy, *DC + Bev* docetaxel + carboplatin + bevacizumab + bevacizumab maintenance therapy

*One patient received only day 1 of cycle 1 of weekly TC therapy

### Surgical outcomes

In the NAC cohort, 152 patients (29%) received exploratory surgery, and the remaining 360 patients (71%) had no surgery before chemotherapy. IDS was applied to 451 of 512 patients (88%) in the NAC cohort, 284 (55%), 107 (21%), and 60 (12%) patients achieved complete surgery (no macroscopic residual tumor), optimal surgery (macroscopic residual tumor < 1 cm), and suboptimal surgery (macroscopic residual tumor ≥ 1 cm), respectively. In the PDS cohort, 167 (39%), 91 (21%), and 170 (40%) patients achieved complete surgery, optimal surgery, and suboptimal surgery, respectively.

### Patient characteristics of the matched cohort

The matched cohort was well-balanced between the NAC and PDS cohorts in terms of various clinical factors, except for histology and total cycle number of chemotherapy (Table 3). The NAC cohort was more likely to have high-grade serous carcinoma (HGSC) than the PDS cohort, and had received significantly more cycles of chemotherapy.

**Table 3:** Patient characteristics (matching cohort)

	NAC cohort (N = 243)	PDS cohort (N = 243)	p
Age (median, range)	58 (51–66)	59 (51–68)	0.630^*^
PS			
0	106 (44%)	102 (42%)	
1	81 (33%)	89 (37%)	
2–	56 (23%)	52 (21%)	0.664^**^
FIGO stage			
IIIA–B	22 (9%)	17 (7%)	
IIIC	156 (64%)	154 (63%)	
IV	65 (27%)	72 (30%)	0.603^**^
Histology			
HGSC	95 (39%)	60 (25%)	
Non-HGSC	148 (61%)	183 (75%)	< 0.001^**^
Ascites			
Paracentesis	55 (23%)	59 (24%)	
Present	117 (48%)	126 (52%)	
Absent	71 (29%)	58 (24%)	0.410^**^
Thrombosis			
None	204 (84%)	205 (84%)	
DVT	23 (10%)	22 (9%)	
PE	16 (7%)	16 (7%)	0.988^**^
Surgery			
Primary surgery	86 (35%)	243 (100%)	
IDS	217 (89%)	4 (2%)	< 0.001^**^
NAC cycle number			
–2	9 (4%)	0	
3	49 (20%)	0	
4	86 (35%)	0	
5	32 (13%)	0	
6–	67 (37%)	0	< 0.001^*^
Adjuvant			
–3	173 (71%)	42 (17%)	
Cycle number			
4	48 (20%)	11 (5%)	
5	8 (3%)	11 (5%)	
6	8 (3%)	115 (47%)	
7–8	1 (0%)	38 (16%)	
9–	5 (2%)	26 (11%)	< 0.001^*^
Total			
3	5 (2%)	42 (17%)	
Cycle number			
4	5 (2%)	11 (5%)	
5	10 (4%)	11 (5%)	
6	63 (26%)	115 (47%)	
7–8	112 (46%)	38 (16%)	
9–	48 (20%)	26 (11%)	< 0.001^*^

*Mann–Whitney U-test, **chi-square test

*NAC* neoadjuvant chemotherapy, *PDS* primary debulking surgery, *HGSC* high-grade serous carcinoma, *DVT* deep venous thrombosis, *PE* pulmonary embolism

### Survival

**Fig. 2:**
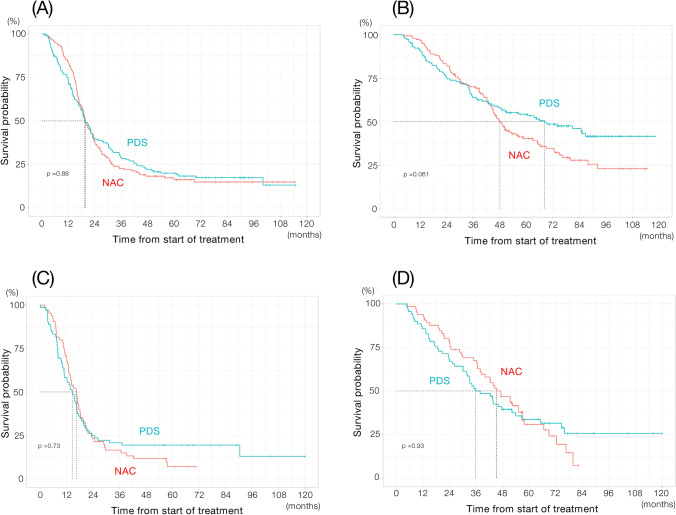
Treatment outcomes according to the International Federation of Gynecology and Obstetrics (FIGO) stage. **A** Progression-free survival of patients with FIGO stage IIIC [neoadjuvant chemotherapy (NAC) cohort: N = 156; primary debulking surgery (PDS) cohort: N = 154]. **B** Overall survival of patients with FIGO stage IIIC (NAC cohort: N = 156; PDS cohort: N = 154). **C** Progression-free survival of patients with FIGO stage IV (NAC cohort: N = 65; PDS cohort: N = 72). **D** Overall survival of patients with FIGO stage IV (NAC cohort: N = 65; PDS cohort: N = 72). Red line: NAC cohort; blue line: PDS cohort

The median follow-up period was 44 months (range: 5–163) in the NAC cohort and 50 (range: 4–120) in the PDS cohort. In patients with FIGO stage IIIC, the NAC and PDS cohorts showed comparable PFS (median PFS: 19.7 vs. 19.4 months, HR: 1.02; 95% confidence interval [CI]: 0.80–1.31, p = 0.88) (Fig. 2A). The NAC cohort had a slightly shorter OS (median OS: 48.1 vs. 68.2 months, HR: 1.34; 95% CI: 0.99–1.82, p = 0.061) than the PDS cohort, albeit not statistically significant (Fig. 2B). Patients with FIGO stage IV in both cohorts had a comparable PFS (median PFS: 16.6 vs. 14.7 months, HR: 1.07; 95% CI: 0.74–1.53, p = 0.73) and OS (median PFS: 45.2 vs. 35.7 months, HR: 0.98; 95% CI: 0.65–1.47, p = 0.93) (Fig. 2C and D).

**Fig. 3:**
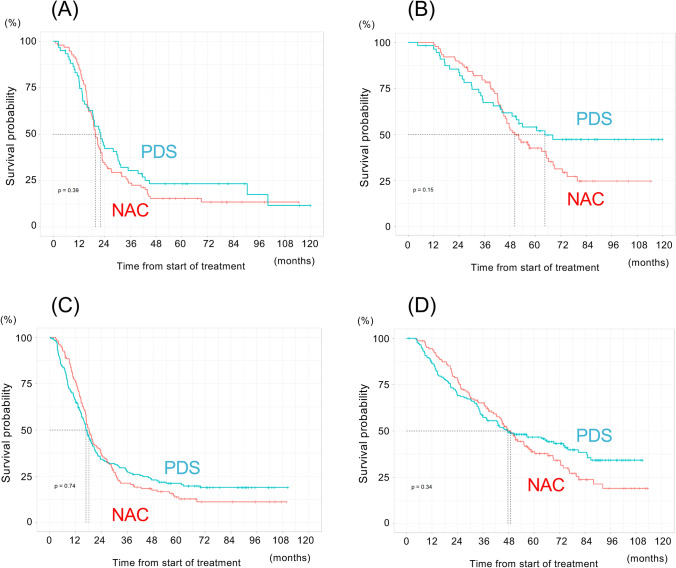
Treatment outcomes according to histological type. **A** Progression-free survival of patients with high-grade serous carcinoma (HGSC) [neoadjuvant chemotherapy (NAC) cohort: N = 95; primary debulking surgery (PDS) cohort: N = 60)]. **B** Overall survival of patients with HGSC (NAC cohort: N = 95; PDS cohort: N = 60). **C** Progression-free survival of patients with non-HGSC (NAC cohort: N = 148; PDS cohort: N = 183). **D** Overall survival of patients with non-HGSC (NAC cohort: N = 148; PDS cohort: N = 183). *Red line* NAC cohort, *blue line* PDS cohort

Patients with HGSC who received NAC or PDS had comparable PFS (median PFS: 19.7 vs. 22.2 months, HR: 1.30; 95% CI: 0.83–2.05, p = 0.34) and OS (median PFS: 50.3 vs. 64.5 months, HR: 1.40; 95% CI: 0.88–2.21, p = 0.15) (Fig. 3A and B). Similar results were found in patients with non-HGSC (median PFS: 18.2 vs. 16.8 months, HR: 1.04; 95% CI: 0.82–1.33, p = 0.74; and median PFS: 49.0 vs. 47.7 months, HR: 1.15; 95% CI: 0.86–1.52, p = 0.34) (Fig. 3C and D).

**Fig. 4:**
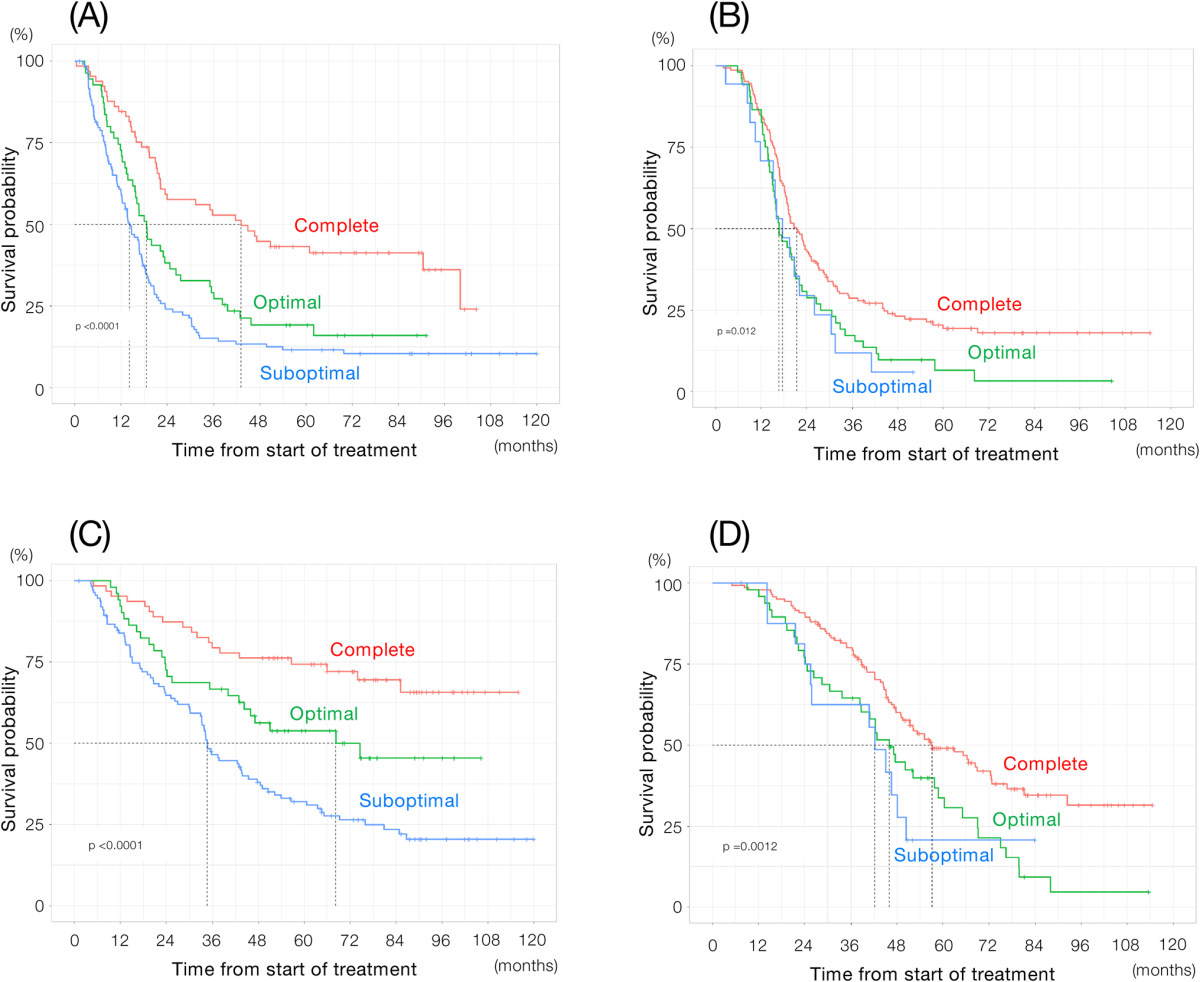
Survival according to the surgical outcome. **A** Progression-free survival in patients who received primary debulking surgery (complete surgery: N = 167; optimal surgery: N = 91; suboptimal surgery: N = 170). **B** Progression-free survival in patients who received interval debulking surgery following neoadjuvant chemotherapy (complete surgery: N = 284; optimal surgery: N = 107; suboptimal surgery: N = 60). **C** Overall survival in patients who received primary debulking surgery (complete surgery: N = 167; optimal surgery: N = 91; suboptimal surgery: N = 170). **D** Overall survival in patients who received interval debulking surgery following neoadjuvant chemotherapy (complete surgery: N = 284; optimal surgery: N = 107; suboptimal surgery: N = 60). *Red line* complete surgery (no macroscopic residual tumor), *green line* optimal surgery (macroscopic residual tumor < 1 cm), *blue line* suboptimal surgery (macroscopic residual tumor ≥ 1 cm)

The median PFS of patients who achieved complete, optimal, and suboptimal surgery following PDS was 43.1, 18.6, and 14.3 months, respectively, whereas that of those who received NAC followed by IDS was 21.5, 16.8, and 17.7 months, respectively (Fig. 4A and B). The median OS of patients who achieved complete, optimal, and suboptimal surgery following PDS was not reached, 68.2, and 34.6 months, respectively, whereas that of those who received NAC followed by IDS was 57.2, 46.0, and 42.2 months, respectively (Fig. 4C and D).

## Discussion

Between 2010 and 2015, more than half of the patients (55%) with FIGO stage III to IV epithelial ovarian cancer received NAC in our study population. The adoption rate of NAC varied widely across institutions from 21 to 67%. Only 39% of patients achieved complete surgery with PDS; in contrast, 55% of patients who received IDS after NAC achieved complete surgery. However, complete surgery with IDS had an effect on survival comparable to that of optimal surgery at PDS. In patients with FIGO stage IV, the PFS and OS were comparable between NAC and PDS. PFS was also equivalent between groups in patients with FIGO stage IIIC; however, the NAC group had a slightly shorter OS (median OS: 48.1 vs. 68.2 months, HR: 1.34; 95% CI: 0.99–1.82, p = 0.061) than the PDS group did.

Disappointing survival data following NAC for advanced ovarian cancer have been reported in many previous studies [1]. Real-world data analysis using propensity score matching in the United States demonstrated that NAC was associated with a shorter OS in stage IIIC disease (HR: 1.40) compared with PDS, but not in stage IV disease (HR: 1.16) [11]. The survival data of said study were very similar to the OS rate we reported, and the OS survival curves in these two cohort studies closely resembled each other. Most patients in both cohorts received a combination of taxanes and platinum. On the other hand, NAC was applied in fewer patients with both stage IIIC (34% vs. 59%) and IV (62% vs. 68%) disease in the United States cohort than in the Japanese cohort in our study. These similarities regarding disappointing survival data validate the concerns on the imprudent application of NAC.

This study confirmed that complete resection with PDS results in the best survival, even in patients with a high tumor burden. The PFS and OS of patients who achieved complete resection with IDS were apparently shorter than those of patients who achieved complete resection with PDS (median PFS: 21.5 vs. 43.1 months, HR: 1.47; 95% CI: 1.23–1.76, p < 0.0001; median OS: 57.2 months vs. did not reach the median, HR: 1.44; 95% CI: 1.21–1.72, p < 0.0001). The survival of patients receiving complete resection with IDS was comparable to that of receiving optimal resection with PDS; the survival curve of patients with complete surgery with IDS also resembled that of those with optimal surgery with PDS. Furthermore, optimal resection with IDS had an effect on survival comparable to that of suboptimal resection with PDS. These results are consistent with those of previous studies, wherein complete cytoreduction after IDS yielded an inferior outcome in terms of median survival compared with PDS almost two years [12, 13].

Inadequate improvement of survival by maximizing tumor debulking with IDS is speculated as the primary cause of the disappointing survival data in NAC. The most important positive effect of PDS is the improvement in the effect of chemotherapy through the removal of poorly perfused tumor portions that receive inadequate doses of chemotherapy and are phenotypically resistant [14]. In contrast, NAC may increase the induction of platinum-resistant clones. A study on recurrent ovarian cancer retreated with platinum-based chemotherapy showed that 88.8% of patients who received NAC were considered platinum-resistant (recurrence within six months) compared with 55.3% in the PDS group (p < 0.001) [15]. Another possible culprit of the shortened survival in the NAC cohort could be that a certain number of patients could not receive IDS because of tumor progression. In this study, 11% of patients who received NAC did not receive IDS. In recent randomized controlled trials (RCTs), 9.5%–15% of patients allocated to the NAC arm did not undergo IDS [5, 6, 16].

The complete reduction rate of patients who received PDS in this study (39%) was significantly higher than those reported in the previous RCTs. The EORTC-55971 trial achieved complete resection in 19.4% of PDS patients; CHORUS, 17%; and JCOG0602, 12% [5, 6, 8]. The participating institutions in our study may have selected patients for PDS more carefully. However, the deliberate choice of patients who were expected to achieve complete resection did not improve survival. Thus, the development of methods to identify patients who are expected to achieve complete surgery is warranted. Recently, exploratory laparoscopy before NAC has been widely used to assess tumor extension and biopsy. The predictive index value score, which was designed to enable an objective assessment of the tumor extent by laparoscopic findings, was reported to be in accordance with the findings of laparotomy with PDS [17, 18].

Our study has three major limitations. Firstly, 59 of 152 patients who received primary surgery included in the NAC group, could not determine the pre-planned goal of primary surgery. Among patients who attempted PDS, those who underwent complete surgery and might have a good prognosis were assigned to the PDS cohort, and those who were unable to undergo complete surgery and might have a poor prognosis were assigned to the NAC cohort. This may have led to underestimation of survival data in the NAC cohort, although correction was performed using the matching methods in the final analysis. Secondly, the chemotherapy regimen differed between the NAC and PDS cohorts. More patients in the NAC cohort tended to receive dose-dense TC therapy than those in the PDS cohort. This may have improved the survival of the NAC cohort. Conversely, patients in the PDS cohort were more likely to receive adjuvant chemotherapy, including bevacizumab, than those in the NAC cohort. Lastly, the effect of the matching procedure might be insufficient. Stage III patients belonging to the PDS cohort may include patients diagnosed preoperatively as stage I or stage II disease and may tend to have a better prognosis. The NAC cohort was more likely to include HGSC than the PDS cohort. In addition, patients belonging to the NAC cohort tended to receive more cycles of chemotherapy than the PDS cohort. We cannot deny the possibility that such a bias that cannot be fully adjusted by matching affects the results.

The role of NAC may be altered according to the regimen used. In the past one to two decades, the standard regimen for advanced epithelial ovarian cancer has been rapidly changing. Alterations in the paclitaxel schedule have been widely explored, with conflicting results [19–21]. Additionally, a PFS benefit from the addition of bevacizumab to paclitaxel and carboplatin was reported in two large RCTs [22, 23]. Recently, four RCTs demonstrated remarkable improvement in PFS with poly adenosine diphosphate-ribose polymerase inhibitor (PARPi) for advanced epithelial ovarian cancer [24]. The results of these trials have changed the regimen applied to advanced epithelial ovarian cancer, resulting in significant change of the position of surgery. Two RCTs, TRUST trial (ENGOT ov33/AGO‐OVAR OP7) conducted by German AGO and SUNNY trial conducted by Shanghai GOG, comparing IDS after three cycles of NAC with chemotherapy after PDS are currently underway [25, 26]. These trials have the primary objective of evaluating whether quality-assured PDS followed by chemotherapy improve survival compared with IDS following NAC in patients with advanced epithelial ovarian cancer. Among them, TRUST trial allows maintenance therapy with bevacizumab and PARPi. The results of these RCTs are expected to clarify the role of NAC followed by IDS under the latest chemotherapy.

More than half of the patients with FIGO stage III to IV epithelial ovarian cancer received NAC between 2010 and 2015. NAC enabled approximately two-thirds of the patients to achieve complete surgery; however, complete surgery with IDS had an effect on survival comparable to that of optimal surgery with PDS. As a result, the strategy using NAC followed by IDS did not improve survival in these patients. Particularly, in patients with FIGO stage IIIC, NAC may be associated with a shorter OS. The easy application of NAC in patients with FIGO stage IIIC epithelial ovarian cancer should be avoided. In the future, we plan to investigate the effects of changes in standard chemotherapy on the application of NAC and patient survival.

## Data Availability

The datasets analyzed in the current study are not publicly available due to privacy reasons, but are available from the corresponding author on reasonable request.
